# Sensitive and molecular size-selective detection of proteins using a chip-based and heteroliganded gold nanoisland by localized surface plasmon resonance spectroscopy

**DOI:** 10.1186/1556-276X-6-336

**Published:** 2011-04-14

**Authors:** Surin Hong, Suseung Lee, Jongheop Yi

**Affiliations:** 1 World Class University (WCU) Program of Chemical Convergence for Energy & Environment (C2E2), School of Chemical and Biological Engineering, Institute of Chemical Processes, Seoul National University, Seoul 151-744, Korea

## Abstract

A highly sensitive and molecular size-selective method for the detection of proteins using heteroliganded gold nanoislands and localized surface plasmon resonance (LSPR) is described. Two different heteroligands with different chain lengths (3-mercaptopionicacid and decanethiol) were used in fabricating nanoholes for the size-dependent separation of a protein in comparison with its aggregate. Their ratios on gold nanoisland were optimized for the sensitive detection of superoxide dismutase (SOD1). This protein has been implicated in the pathology of amyotrophic lateral sclerosis (ALS). Upon exposure of the optimized gold nanoisland to a solution of SOD1 and aggregates thereof, changes in the LSPR spectra were observed which are attributed to the size-selective and covalent chemical binding of SOD1 to the nanoholes. With a lower detection limit of 1.0 ng/ml, the method can be used to selectively detect SOD1 in the presence of aggregates at the molecular level.

## Introduction

Many proteins are capable of causing immune reactions, and the presence of protein aggregates has been identified as an important factor leading to the lowering of immune tolerance [1-3]. For this reason, the precise detection of proteins in the presence of other formulations with high sensitivity and specifity is essential for disease diagnostics, drug screening, and other applications [4,5]. The most widely used method for the detection and analysis of proteins and their aggregates in formulations is size exclusion chromatography (SEC) [5]. Although useful for the determination of molecular-weights of proteins, its application to the quantitative determination of protein concentrations is more difficult. Another spectroscopic techniques involving the use of light scattering techniques [6] and Fourier transform infrared spectroscopy (FTIR) [7,8] are also frequently used for this purpose. Light scattering methods have been used to calculate the mean hydrodynamic radius of protein aggregates and to characterize molecular distribution of protein aggregates. However, high concentrations of protein solutions are needed to accomplish this. As a result, the technique has limitations involving erroneous interpretations and a lack of sensitivity at low concentrations. Although FTIR method has also been used for the determination of changes in protein secondary structure, it is still difficult to quantitatively analyze and differentiate between protein concentrations in aggregates. In order to overcome these limitations and enhance sensitivity, optical methods such as fluorescence spectroscopy have been used [9]. However, strong background or the quenching of spectroscopic signals resulting from the use of labeling dyes has been reported [10,11]. In this regard, label-free optical methods have been developed and localized surface plasmon-based metallic nanomaterials represent a promising alternative for achieving high sensitivity, and selectivity at low concentrations.

The optical properties of metallic nanomaterials arise from localized surface plasmon resonance (LSPR), which is caused by the collective oscillation of surface conduction electrons by light [12-15]. Changes in the peak intensity and wavelength of plasmon spectra, which are caused by variations in refractive index as the result of the binding of molecules to the metal nanomaterials, are optically detectable parameters that have found use in chemical and biosensensor devices [16-19]. Currently, due to the potential for impacting screening in medical and environmental applicabilities, LSPR sensing systems would be more attractive. However, further improved sensitivity and accuracy of the devices are required, so that the development of a novel nanostructure design with special optical properties for high sensitivity and selectivity has become a priotiry.

Here, we propose a highly sensitive and molecular size-selective detection method for a protein in the presence of its aggregate by utilizing a heteroliganded gold nanoisland on a transparent glass substrate. As a proof-of-concept test, the superoxide dismutase (SOD1) protein was selected for the sensitive and molecular size-selective detection between this protein and aggregates derived from it. SOD1 is a well-known, highly stable dimeric enzyme that catalyzes the dismutation of super radicals to hydrogen peroxide and molecular oxygen. Its aggregated structure in motor neurons is associated with amyotrophic lateral sclerosis (ALS), a neurodegenerative disease [20,21].

The method focused on several significant factors. First, heteroliganded nanoholes were fabricated on gold nanoislands for protein separation based on its physical dimensions in the presence of aggregates. Second, the detection method is based on sensitive changes in the local dielectric environment of modified gold nanoislands, which are caused by covalent chemical interactions between the proteins and the active sites of nanoholes. Third, the transduction system was modified for application to a chip-based detection method. This would premit the fabrication of materials using off-the-shelf materials with high stability, which would be applicable to simple, low-cost diagnostics. The combination of these factors would result in the more sensitive, molecular size-selective, and simpler method for the determination of a protein.

## Materials and experiments

### Materials

3-Mercaptopionicacid (MPA, Sigma-Aldrich Korea Ltd.), decanethiol (DT, Sigma), *N*-(3-dimethylaminopropyl)-*N*-ethylcarbodiimide hydrochloride (EDC, Sigma-Aldrich Korea Ltd.), and *N*-hydroxysuccinimide (NHS, Sigma-Aldrich Korea Ltd.) were used as received. A piranha solution (70% sulfuric acid (H_2_SO_4_, Fisher Scientific Korea Ltd.) and 30% hydrogen peroxide (H_2_O_2_, Sigma-Aldrich Korea Ltd.)) was used to clean the glass substrates on which gold nanoisland were fabricated (*caution*: piranha solutions should be handled with extreme care).

### Fabrication of the gold nanoisland substrate

The gold nanoisland was prepared by the thermal evaporation on a glass substrate (0.8 × 7.0 cm) in a vacuum at a temperature of 65°C. The substrate was then annealed at 200°C for 5 h to produce more stable and ordered gold nanoislands. Two different types of gold nanoislands (1- and 5-nm thickness) modified with 3-mercaptopionicacid (MPA, short chain length) and decanethiol (DT, long chain lengh, relatively) were prepared on the glass substrate. A two-step procedure was used in preparing the functionalized and activated nanoisland. In the first step, a mixed self-assembled monolayer (SAM) of MPA and DT on the gold nanoisland was prepared by treatment with different volumetric ratios of 1 mM ethanolic MPA and DT solutions overnight. In the second step, the COOH groups of MPA were activated to reacive esters by reaction with NHS and EDC [22,23], followed by reaction with an amine-terminated SOD1 protein to covalently tether the protein to the surface of the gold nanoisland.

### SOD1 protein expression and purification

The wild-type human SOD1 gene was cloned into the pET23b (+) (Novagen) vector and the protein was expressed in *E. coli*. Cultures were induced by the addition of 0.5 mM isopropyl b-D-thiogalactopyranoside for 3 to 6 h at 30°C, and the cells were then lysed by sonication in a buffer containing 150 mM NaCl, 50 mM Tris-HCl (pH 8.0), 0.1 mM EDTA, 1 mM dithiothreitol (DTT), and 1 mM phenylmethylsulfonyl fluoride (PMSF). Proteins were eluted with a linear gradient of ammonium sulfate (0.75-0 M) in 50 mM sodium phosphate (pH 7.0), 150 mM NaCl, 0.1 mM EDTA, and 0.25 mM DTT. Wild-type SOD1 was released with high specificity from the column at concentrations between 1.3 and 0.8 M ammonium sulfate. The mutant SOD1 (A4V) was further purified by gel permeation chromatography on a Superdex 200 column (GE healthcare) in 50 mM sodium phosphate buffer (pH 7.0, 150 mM NaCl). Zn and Cu were removed from the protein, to give apo-type SOD1.

### Preparation of SOD1 aggregates

The purified wild-type apo-SOD1 molecules were diluted with an acidic phosphate-buffered saline (PBS) solution (pH 5.4) to a concentration of 0.1 mg/ml. SOD1 aggregates were prepared by treatment with a solution containing 20% (v/v) trifluoroethanol (TFE) [24,25].

### Optical absorption spectroscopy

Optical absorption spectroscopy measurements were performed in a spectrophotometer (HP 8453) using 1-cm path-length quartz cuvettes. Spectra were collected over the 400-800-nm wavelength range.

## Results and discussion

### Principle of heteroliganded gold nanoisland-based sensing platform

**Figure 1:**
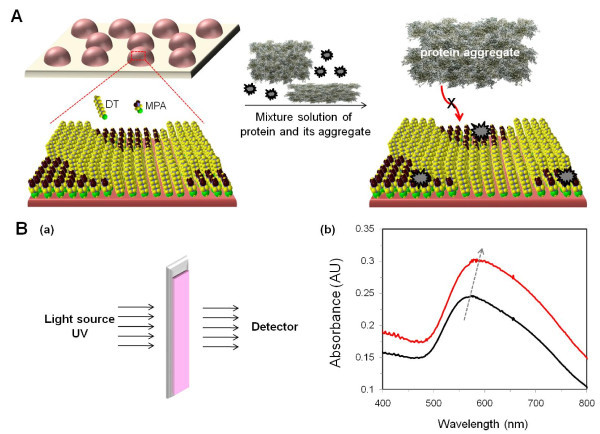
**(A) Schematic view of a heteroliganded gold nanoisland for the sensitive and molecular size-selective detection of a protein**. The enlarged schematic diagram shows the surface morphology of a heteroliganded gold nanoisland and the binding principle of a protein in the presence of its aggregates. **(B) **(a) The detection method via chip-based localized surface plasmon resonance spectroscopy. (b) The before (black) and after (red) absorption spectra of the gold nanoislands after a 1-h exposure to a 0.1 mg/ml solution of SOD1.

We examined the feasibility of a chip-based gold nanoisland sensor prepared for the sensitive and molecular size-selective detection of a protein. A schematic view of the heteroliganded gold nanoisland and the basic scheme underlying the sensor operation are shown in Figure 1. The gold nanoisland was prepared by thermal evaporation on a transparent substrate. After annealing procedure of the surface, dimensions of the gold nanoislands were in the range of 40-80 nm in diameter [26,27]. The binary SAM containing MPA and DT was fabricated on the gold nanoislands. Because the two thiol derivaties, MPA and DT, have different hydrophilicities as well as different chain lengths, the nanometer-scale phase separation on the surface would form a binary mixture due to *ω*-functional group interaction [28-31]. The mole fractions of these molecules on the gold nanoisland in the fabrication of the mixed SAM were optimized so as to maximize the sensitivity of the method. The phase separated and nanometer-scaled MPA domains in the mixed SAM induce the formation of nanoholes [32-35], which would play an important role as effective binding sites of native SOD1 compared to aggregates. The dimensions of SOD1 aggregates cultured by TFE condition for 4 weeks was determined to be in the several hundred nanometer scales to the micrometer scales [36]. Because of this, it is not possible for them to enter the MPA nanoholes.

The heteroliganded gold nanoisland substrate with the mixed SAM was then partially activated (the area of MPA) to receive esters to covalently tether the protein to the surface of the gold nanoisland. The hydrophobic moieties of DT functions allow the proteins to approach the activated receptor more easily, resulting in the binding of the protein to the activated MPA sites via carboimide coupling to protein-free anime moieties. We hypothesize that the surface of the gold nanoisland has a golf ball-like morphology with nanoholes, which would permit the sensitive and size-selective detection of a protein over an aggregated species in a variety of formulations. (Figure 1A).

The binding interaction between the proteins and the nanoholes on the gold nanoisland surface caused distint shifts in the LSPR peak, as detected by UV-visible absorption spectrometry. Figure 1B shows the chip-based detection system and representative spectra of LSPR for the SOD1 protein for a concentration of 0.1 mg/ml. The optical properties of LSPR in a gold nanoisland can be utilized to transduce the optical signal change in their absorbance spectrum. Only the gold nanoisland glass surface shows a plasmon resonance peak centered at 550 nm wavelength, which could confirmed that the island shape grew a near-hemisphere shape [37], and the correspoding color of the substrate is pink-red. When the glass substrate is exposed to the protein solution, a remarkable change in the maximum absorption for the surface plasmon resonance peak was observed. From the spectra, the detection method utilizing a heteroliganded gold nanoisland and the optical properties of LSPR permit the concentration to be determined at an extremly low level-concentration of protein in a homogeneous solution. In addition, the method permits the protein to be separated from its macrospecies (protein aggregates) that are derived from the protein, and predict the ratio of native form to the concentration of aggregates. Finally, the method permits the basic parts to the readily fabricated from off-the shelf materials that are reasonably stable, and its subsequent application to simple and low-cost diagnostics.

### Optimization of heterolihanded gold nanoisland for the high sensitivity

For the sensitive detection of a protein, the thickness of the gold nanoisland and the ratio of the two ligand motifs were optimized. The gold nanoisland glass substrates were fabricated by thermal evaporation with thickness from 1 to 10 nm. For thicnkesses of over the 7 nm, the color of the substrate changed from red to purple and bluish purple, i.e., the distance between the gold nanoislands appeared to be close and aggregated. When the thickness was over 10 nm, no gold nanoislands were formed, but a thin gold flat film was formed (data not shown). Thus, we selected gold nanoisland substrates with thicknesses of 1 and 5 nm for use and the sensitivity of these substrates were evaluated based on the response traces at the fixed maximum peak of absorbance.

On the two types of gold nanoisland substrates, the mole fraction between DT (Ӽ_DT_) and MPA (Ӽ_MPA_) was varied as 1:1, 10:1, 50:1, 100:1, and 200:1 (Ӽ_MPA _is 0.5, 0.1, 0.02, 0.01, 0.005, respectively, where Ӽ_MPA _+ Ӽ_DT _= 1). The surface property of these different ratios of heteroligands was supported by water contact angle measurements. In the case of a greater ratio of DT, the hydrophobicity of the surface increases and the angle for each substrate was determined to be 42.1°, 43.3°, 46.0°, 48.5°, and 50.1° respectively.

**Figure 2:**
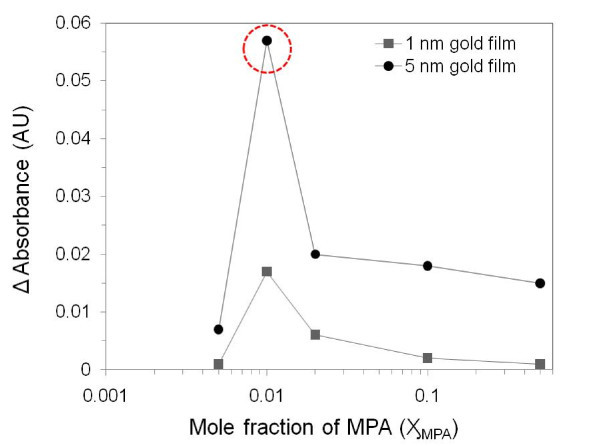
**Changes in the maximum absorbance peak as a function of mole fractions of MPA over DT in binary mixed SAM (Ӽ**_**MPA **_**+ Ӽ**_**DT **_**= 1)**.

Figure 2 shows changes in absorbance as a function of the mole fraction of MPA (semi-log scale) when nanoisland substrates with two different thicknesses (1, 5 nm) were exposed to an aqueous SOD1 protein solution (0.1 mg/ml) for 1 h, and then allowed to equilibrate after exposure to dry air, respectively. Among the different substrates, the changes in absorbance of the 5-nm nanoisland substrates were superior than that of the 1-nm substrates. This can be attributed to the more extensive coverage of gold nanoisland in the case of the 5-nm substrate. This would result in a stronger absorption efficiency of the electromagnetic field proportional to the imaginary part of polarizability [37]. A plot of sensitivity as a function of ratio shows that the sensitivity increases with the mole fraction, reaching a maximum change for a Ӽ_MPA _value of 0.01, and then drastically decreases. Therefore, the optimized gold nanoisland glass substrate was used to examine the sensitivity of the method for the detection of various concentrations of SOD1.

### Sensitive detection of SOD1 protein

**Figure 3:**
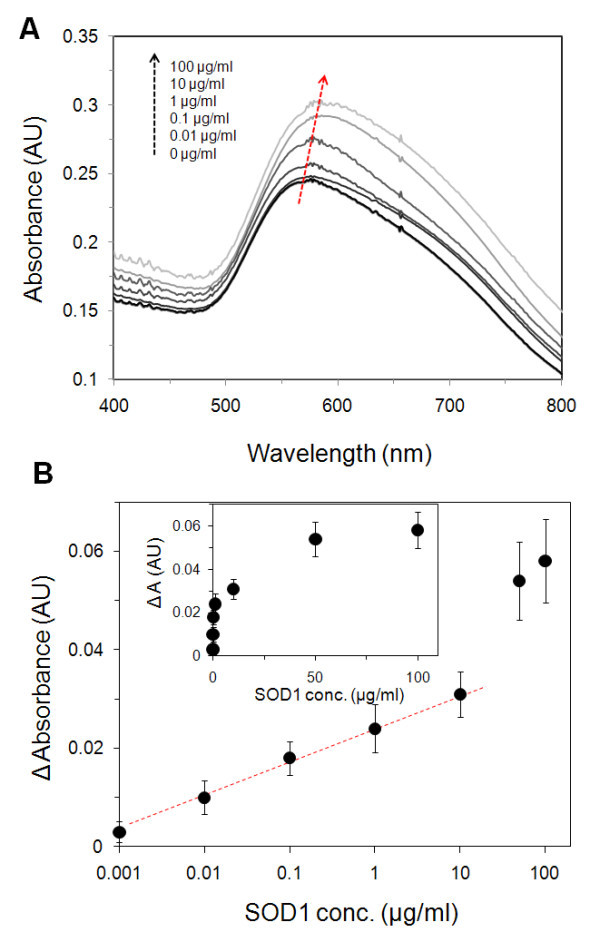
**(A) Representative absorption spectra of SOD1 concentration-dependent response**. (B) Changes in the maximum absorbance peak as a function of SOD1 concentration.

To explore the sensitivity of the method using a homogeneous protein solution, changes in maximum absoprtion at the surface plasmon peak after a 1-h exposure were determined for SOD1 protein concentrations in the range of 1.0 to 0.1 mg/ml. Figure 3A shows representative absorption spectra for the SOD1 concentration-dependent response. When the gold nanoisland substrate is exposed to an SOD1 solution, the peak position is red and shifted upward, compared to the spectra of the native gold nanoisland substrate. Overall in these experiments, the wavelength shift at the centroid peak (the signal of red shift) was not sensitive at low concentrations of SOD1. Thus, the change in absorption near the surface plasmon peak is the preferred optical signal. As seen in Figure 3B, upon increasing the protein cencentration, the changes in the absorption also increase. Concerning signal noise, the limit of detection was determined to be 1.0 ng/ml. It should be noted that there is linear increase in the absorbance changes for the concentration range of 1.0 ng/ml to 10 μg/ml (semi-log scale). At higher concentrations, the binding of SOD1 became saturated, leading to a nonlinear response and a plateau for concentrations in excess of 50 μg/ml. The reason for the non-linear response at higher SOD1 concentrations is that the binding site of the gold nanoisland was nearly saturated with SOD1 protein. That is, the operational amplitude of absorption change (Δ*A*) increases in proportion to the SOD1 concentration, reaching a limiting value (Δ*A *= 0.058). The calibration curve also shows that the heteroliganded gold nanoisland can detect roughly a five- to tenfold lower concentration of the protein than other metallic nanoparticle systems [38,39].

### Size-selective detection of SOD1 in the presence of its aggregate

For the molecular size-selective detection of a protein formulation, a mixture of solutions of native SOD1 (100 μg/ml) and its aggregates (the volumetric ratio between them was 1:1, 1:5, 1:10, 1:20.) were exposed to the gold nanoisland glass substrate and changes in the absorption spectra were measured. The aggregated SOD1 protein formulation was verified using circular dichroism (CD) spectroscopy (Data not shown; our previous published data indicates that significant differences in CD spectra were observed between SOD1 incubated with TFE and freshly prepared SOD1. The secondary structure of native SOD1 is mainly comprised of β-sheets (60%) and random coils (30%) with the remainder being α-helixes. Changes in the secondary structure of SOD1, i.e., a decrease in the β-sheet and α-helix content of SOD1 together with an increase in the random coil content, indicate the formation of aggregates [36].)

**Figure 4:**
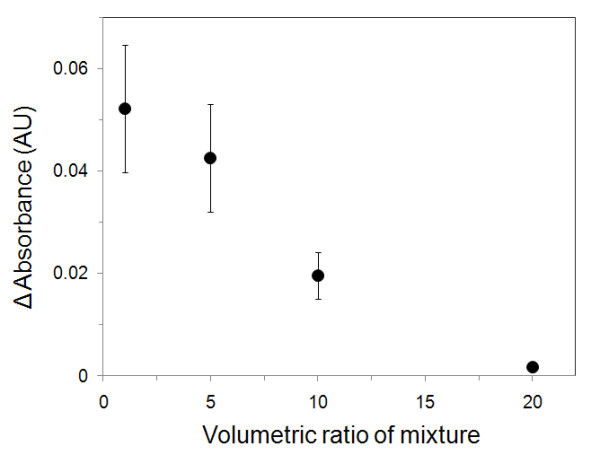
**Changes in the maximum absorbance peak as a function of the volumetric ratio of a mixed SOD1formulation**.

Upon exposure to the solution of native SOD1 and aggregates thereof, the nanometer-scaled MPA domains (nanoholes) in the mixed SAM can act as a molecular sieve, the SOD1 that are present into the nanoholes then selectively bind to the activated sites on MPA. Figure 4 shows changes in the maximum absorption peak as a function of the volumetric ratio of SOD1 formulations. As the ratio of SOD1 aggregates increases, the change in absorbance peak decreases, and this dependence was dependent on the amount of native SOD1 initially present in the solution. Finally, when the ratio was 1:20, the spectra did not change further. This result suggests that the existance of large amounts of aggregates in the mixture solution may interfere with the an aproach of the native SOD1 to the nanoholes. From the results obtained herein, the heteroliganded gold nanoisland and the detection method permit us to predict the ratio of a protein and macrospecies derived from a protein. Moreover, the method has the portntial for being appplied to other protein fomulations and has considerable potential for use in chip-based diagnoses.

## Conclusions

In conclusion, we describe a highly sensitive and molecular size-selective detection method for determining proteins with heteroliganded gold nanoisland on an optically transparent substrate via LSPR detection by UV-visible absorption spectrometry. To achieve this, two specific ligands of MPA and DT with different chain lenghs were used to fabricate nanoholes on the gold nanoisland. The thickness of the gold nanoisland and the ratio of heteroligand were optimized so as to maximize the sensitivity of the method. The area of MPA was locally activated to reactive esters to permit the binding of the protein. Upon exposure the optimized gold nanoisland to an SOD1 solution, the limit of detection was determined to be 1.0 ng/ml, which is significantly more sensitive than other existing optical methods. Since the nanoholes may act as a sieve by virtue of their physical dimensions, the method is also molecular size-selective for SOD1 in the presence of its aggregates. Thus, this optical spectroscopic method using heteroliganded gold nanoislands is potentially useful for the sensitive detection of small biomolecules and the molecular size-dependent screening of formulations that contain them.

## Abbreviations

MPA: 3-mercaptopionicacid; ALS: amyotrophic lateral sclerosis; CD: circular dichroism; DT: decanethiol; FTIR: Fourier transform infrared spectroscopy; LSPR: localized surface plasmon resonance; SAM: self-assembled monolayer; SEC: size exclusion chromatography; SOD1: superoxide dismutase.

## Acknowledgements

This study was supported by a Grant No. 101-081-032 from the Ministry of Environment, Korea, and WCU (World Class University) program through the Korea science and Engineering Foundation funded by the Ministry of Education, Science and Technology (400-2008-0230).

## Competing interests

The authors declare that they have no competing interests.

## Authors' contributions

SH carried out the design of the study, fabricated the film, performed the sensing analysis and drafted the manuscript. SL participated in the fabrication of the film. JY conceived of the study, and participated in its design and coordination. All authors read and approved the final manuscript.
